# An Improved Mampel Model of the Non-Isothermal Crystallization Kinetics of Fiber-Reinforced Thermoplastic Composites

**DOI:** 10.3390/ma17194747

**Published:** 2024-09-27

**Authors:** Zengrui Song, Huiming Ning, Feng Liu, Ning Hu, Youkun Gong

**Affiliations:** 1 College of Aerospace Engineering, Chongqing University, 174 Shazheng St., Shapingba District, Chongqing 400044, China; songzengrui@live.com (Z.S.); ninghu@cqu.edu.cn (N.H.); liufeng@cqu.edu.cn (F.L.); 2 School of Mechanical Engineering, Hebei University of Technology, Tianjin 300401, China; 3 Chongqing Polycomp International Corporation, Chongqing 300401, China; gongyoukun@cpicfiber.com

**Keywords:** thermoplastic composites, non-isothermal, crystallization kinetics

## Abstract

Fiber-reinforced thermoplastic composites (FRTPs) are gaining increasing attention and widespread use in engineering applications due to their high specific strength and stiffness, excellent toughness, and recyclability. The mechanical properties of these composites are closely tied to their crystallization process, making it crucial to accurately describe this phenomenon. Existing theoretical models for analyzing the non-isothermal crystallization of thermoplastic composites often face challenges relating to the complexity of obtaining multiple parameters and the difficulty of achieving a final relative crystallinity of 1. To address these issues, this paper introduces a novel functional form of the crystallization rate parameter *K*(*T*), tailored for engineering applications, and proposes an improved Mampel model. This model assumes *K*(*T*) to be zero before the onset of crystallization and also to be linearly dependent on temperature thereafter, ensuring that the final relative crystallinity reaches 1. The model requires only two easily accessible parameters: the initial crystallization temperature (*T*_s_) and the linear slope (*k*). The simplicity of the model makes it particularly well suited to engineering applications. This provides a straightforward and effective tool for describing the non-isothermal crystallization kinetics of fiber-reinforced thermoplastic composites.

## 1. Introduction

Thermoplastic composites have emerged as a promising direction in composite material development owing to their exceptional mechanical properties, recyclability, unlimited prepreg storage life, and short production cycles. The thermo-stamping process is particularly well suited for the mass production of small- to medium-sized special-shaped parts made from these materials [1]. The mechanical properties of stamped thermoplastic composites are closely linked to the crystallization process of the thermoplastic matrix [2,3,4]. Consequently, an accurate kinetic model of non-isothermal crystallization is crucial for regulating mechanical properties and optimizing the process parameters of thermoplastic composites [4,5,6,7].

The research on the crystallization of composites primarily encompasses the exploration of crystallization mechanisms and crystallization kinetics. In the realm of crystallization mechanism research, Ruan et al. [8] constructed a multi-scale model that correlates macroscopic temperature with microscopic crystal morphology, employing a coupled finite volume method and pixel coloring algorithm to capture the crystallization morphology. The impacts of the cooling rate, the initial temperature, and the nucleation rate per unit fiber area were investigated. Fang et al. [9] proposed a novel crystallization kinetics model of transcrystalline, applying it to simulate the crystallization behavior of short carbon fiber-reinforced polymer composites. The simulation results, in conjunction with the pixel coloring method [8] and differential scanning calorimetry (DSC) results, provided insights into both isothermal and non-isothermal crystallization processes [10].

Recent studies of composite crystallization kinetics often relied on established models, such as the modified Avrami model [11,12,13,14,15,16,17,18], the Ozawa model [15,16,17,18,19,20], the Mo model [14,15,16,17,18,21,22], and the Nakamura model [4,23,24,25]. For instance, Sun et al. examined the non-isothermal crystallization of continuous glass fiber-reinforced PEEK composites using the modified Avrami model [13]. Wu et al. explored the non-isothermal crystallization kinetics of PA66 fiber-reinforced PA6 composites, deriving parameters for the modified Avrami, Ozawa, and Mo models [17]. Kugele et al. utilized the Nakamura model to analyze the non-isothermal crystallization of continuous carbon fiber-reinforced PA6 composites, achieving a good fit between model data and experimental results [24]. Farjas et al. [26] provided a comprehensive overview of various crystallization kinetics models, including the KJMA model [27], the Mampel model [28], and the Šesták–Berggren model [29]. They also developed a method for obtaining approximate solutions for the evolution of single-step transformations under non-isothermal conditions.

The pivotal aspect of modeling crystallization kinetics relates to the functional form of the crystallization rate parameter *K*(T), which varies across different models. Among these, the Hoffman–Lauritzen equation [30] and the Ziabicki equation [31] are commonly employed. Pérez-Martín et al. analyzed the non-isothermal crystallization of CF/PEKK composites using a modified Nakamura model and the Hoffman–Lauritzen equation [23]. Dörr et al. integrated the Ziabicki equation into the Nakamura model to simulate the punching process of composites [25]. Kulkarni et al. utilized a modified Nakamura–Ziabicki model to describe the crystallization kinetics of CF/PA6 composites and integrated this model into the commercial finite element software COMSOL Multiphysics^®^. The evolution of crystallinity in the laminate is simulated for the process-relevant mold, laminate temperatures, and laminate thicknesses [32]. Huang et al. expanded the parameters *K*_g_ and U* in the Hoffman–Lauritzen equation using Vyazovkin’s method and demonstrated that Nylon 6/PEGMA had higher *K*_g_ and U* values than Nylon 6 [33]. Guo et al. employed the Hoffman–Lauritzen equation to elucidate the crystallization rate parameter *K*(*T*) in the non-isothermal crystallization of polyamide 6/halloysite nanocomposites and analyzed the differences between the crystallization of the composites and PA6 [34].

Although significant progress has been made in the study of non-isothermal crystallization kinetics of composites, existing theoretical models often encounter challenges, particularly with the complexity of obtaining multiple parameters. Additionally, during the data processing of the non-isothermal crystallization of the composites, it was found that the value of *K*(*T*) might not consistently align with the Hoffman–Lauritzen or Ziabicki expression, especially towards the end of the crystallization process. The *K*(*T*) values derived from these equations frequently appeared lower than those from the experimental data, resulting in a phenomenon where the relative crystallinity of certain cooling processes could not reach 1, even when the crystallization rate reached 0. This was a notable discrepancy from actual observations. To address these issues, in this paper, we developed a novel functional form of the crystallization rate parameter *K*(*T*) and proposed an improved Mampel model [26,28]. The improved model ensures that the final relative crystallinity reaches 1 and requires only two easily accessible parameters, making it particularly suitable for engineering applications.

This paper is structured into four sections. Section 2 introduces the improved Mampel model based on the piecewise-linear function of *K*(*T*) and the non-isothermal crystallization of CF/PA6. Section 3 applies the model to the non-isothermal crystallization process of PA6 and other reported composites, analyzing the model’s applicability. Section 4 analyzes the parameters of the model. The final section summarizes the advantages and disadvantages of the model.

## 2. The Improved Non-Isothermal Crystallization Kinetic Model for Thermoplastic Composites

### 2.1. Introduction to Non-Isothermal Crystallization Kinetics Models

The crystallization kinetics model is employed to illustrate the relationship between relative crystallinity and temperature history. The study of isothermal crystallization kinetics of polymers began with Avrami, who proposed the isothermal crystallization kinetics model [35]:

(1) $$X=1-\exp\left(-kt^{n}\right)$$

where *k* represents the crystallization rate constant, *t* denotes time, *n* is the Avrami index, and *X* stands for the relative crystallinity. The latter is defined in Equation (2):

(2) $$X=\frac{\int_{T_{0}}^{T}\left[dH_{c}/dT\right]dT}{\int_{T_{0}}^{T_{\infty }}\left[dH_{c}/dT\right]dT}$$

where *dH_c_* is the enthalpy of crystallization released during an infinitesimal temperature interval *dT*.

The Avrami model was modified by Jeziorny [11], Ozawa [36], and Nakamura [37], and the models they produced are widely utilized in non-isothermal crystallization studies. Nakamura assumed that the number of activated nuclei remains constant and extended the Avrami theory to accommodate crystallization under non-isothermal conditions:

(3) $$X=1-\exp\left[-{\left(\int_{0}^{t}K(T)dt\right)}^{n}\right]$$

By differentiating and rearranging Equation (3), we obtain the differential form of the Nakamura equation:

(4) $$\frac{dX}{dt}=nK(T)(1-X){\left[-\ln(1-X)\right]}^{\frac{n-1}{n}}$$

The Hoffman–Lauritzen equation is often employed to illustrate *K*(*T*) [30]:

(5) $$K=K_{0}\cdot \exp\left(\frac{-U^{*}}{R(T-T_{\infty })}\right)\exp\left(\frac{-K_{g}}{T\cdot \Delta T\cdot f}\right)$$

where *K*_0_ is a coefficient; U* can be assigned a “universal” value of 6284 J/mol; *R* denotes a gas constant of 8.314 J/(mol·K); *T* represents the absolute temperature, *T*_∞_ = *T*_g_ − 30; *T*_g_ denotes glass transition temperature, △*T* = *T*_g_ − *T*; *T*_m_ stands for the equilibrium melting point; and *f* = 2*T*/(*T*_g_ − *T*_m_) serves as a correction factor to accommodate for the reduction in the latent heat of fusion with decreasing temperature.

**Figure 1:**
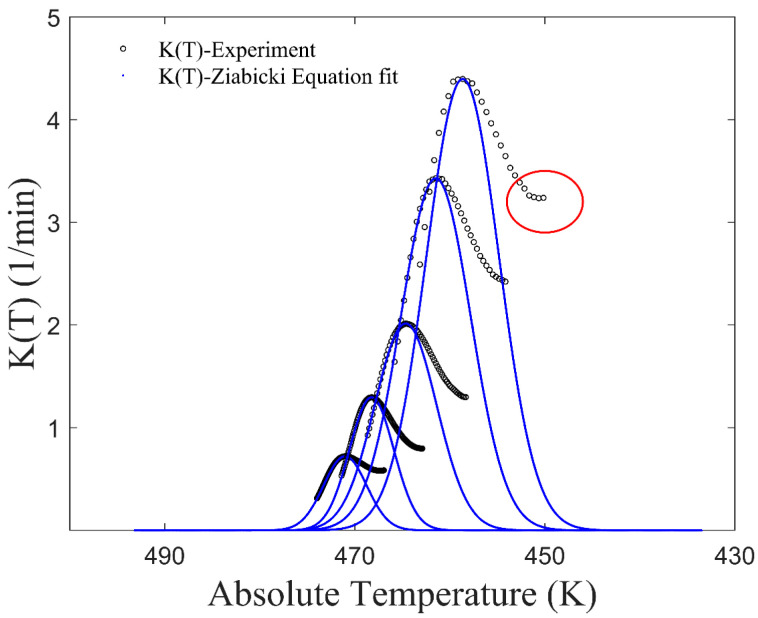
*K*(*T*) of CF/PA6 in Nakamura model.

Considering the intricacies involved in data processing using the Hoffman–Lauritzen equation, Kugele et al. [24] adopted the Ziabicki equation, where *K*(*T*) is illustrated by a curve that resembles a Gaussian function [31]:

(6) $$K(T)=K_{\max}\cdot \exp\left(\frac{-4\ln2{\left(T-T_{\max}\right)}^{2}}{D^{2}}\right)$$

where *K*_max_ represents the maximum value of *K*(*T*); *T*_max_ is the temperature at which *K*(*T*) equals *K*_max_, *D* = 2(*T*_1/2max_ − *T*_max_); and *T*_1/2max_ is the temperature when *K*(*T*) is equal to *K*_max_/2 [32]. All the parameters are contingent on the cooling rate. Nevertheless, the Ziabicki equation may not align closely with *K*(*T*) values obtained from experimental data, particularly towards the end of crystallization. As shown in Figure 1, the disparity between the model and experiment is denoted by a red circle. Obviously, the model data fall below the experimental data, potentially leading to the relative crystallinity failing to reach 1, if the absolute value of the integral in the power function term in Equation (3) is insufficiently large.

### 2.2. The Improved Mampel Model Based on Piecewise-Linear K(T)

In addition to the Nakamura model (Equation (4)), Equation (7) is also utilized to describe the crystallization process [26].

(7) $$\frac{dX}{dt}=K(T)f(X)$$

where *f*(*X*) = 1 − *X* is a commonly used function form [28]. The differential form of the crystallization kinetics model can be illustrated as Equation (8):

(8) $$\frac{dX}{dt}=K(T)(1-X)$$

The integral form of the model can be described as follows:

(9) $$X=1-\exp\left(-\int_{0}^{t}K(T)dt\right)$$

By combining Equations (8) and (9), we obtain the following:

(10) $$\frac{dX}{dt}=K(T)\exp\left(-\int_{0}^{t}K(T)dt\right)$$

To analyze the non-isothermal crystallization kinetics, DSC tests were performed on CF/PA6. The non-isothermal crystallization and melting processes were conducted by heating the CF/PA6 samples from 30 to 280 °C at a rate of 20 °C/min. The samples were held at 280 °C for 10 min to eliminate the thermal history before cooling at specified rates. The samples were then cooled to 30 °C at constant cooling rates of 2.5, 5, 10, 20, and 30 °C/min. All tests were carried out in a pure nitrogen atmosphere.

**Figure 2:**
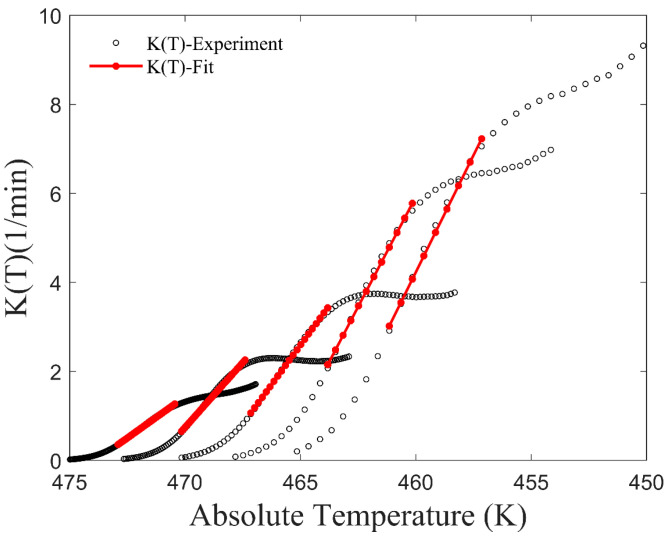
*K*(*T*) of CF/PA6 in the Mampel model and the fitted lines of a certain stage.

First, data sets of temperature *T* and crystallinity *X* under different cooling rates were obtained using the definition of *X* (Equation (2)). Subsequently, data sets of *T* and d*X*/d*t* under different cooling rates were easily obtained via calculation. Then, the values of *K*(*T*) under different cooling rates were calculated using Equation (8), as depicted by the black circle in Figure 2.

When considering the relationship between *K*(*T*) and *T*, it is assumed that the function form is linear during the first half of the crystallization phase. *K*(*T*) data, where the relative crystallinity ranges from 0.1 to 0.6, were selected for linear fitting. The corresponding fitted data are shown by the red line in Figure 2. Prior to the onset of crystallization (high-temperature region), the value of *K*(*T*) is considered to be zero. *T*_s_ is the temperature at which crystallization begins. It is assumed that the crystallization rate parameter *K*(*T*) is linearly related to temperature after *T*_s_. To avoid the aforementioned issue where the relative crystallinity of the model cannot reach 1, *K*(*T*) at the end of the crystallization stage is assumed to be linear with the temperature being as before. The linear slope is denoted as *k*, and the new function form of *K*(*T*) is as follows:

(11) $$K(T)=\left\{\begin{array}{cc} 0 & T\geq T_{s}\\ k\left(T-T_{s}\right) & T<T_{s} \end{array}\right.$$

**Figure 3:**
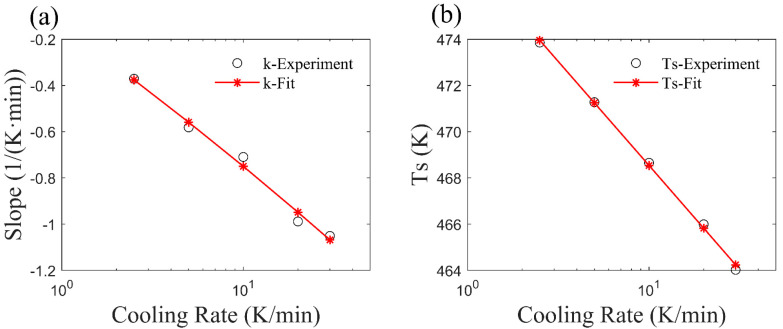
Variation of parameter with cooling rate. (**a**) Variation in *k*. (**b**) Variation in *T*_s_.

The slope values *k* obtained at different cooling rates are shown by black circles in Figure 3a, and the variation in *T*_s_ with the cooling rate is presented in Figure 3b. The adopted fitting function form is shown in Equation (12), where *a*, *b*, *c* and *d* are the material-related parameters, *φ* is the cooling rate, and *φ*_1_ and *φ*_2_ are the minimum and maximum cooling rates in the test. It can be observed that both the slope *k* and the initial crystallization temperature *T*_s_ are significantly influenced by the cooling rate.

(12) $$\left\{\begin{array}{c} k=a\log\left(\varphi \right)+b\\ T_{s}=c\log\left(\varphi \right)+d \end{array}\;\varphi \in \left[\varphi_{1},\varphi_{2}\right]\right.$$

**Figure 4:**
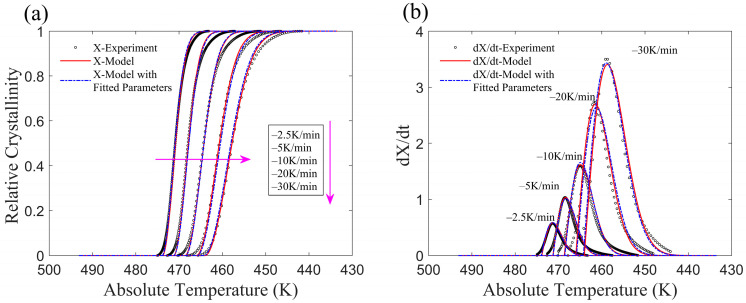
Comparison between Model and experiment of CF/PA6. (**a**) Relative crystallinity of CF/PA6. (**b**) Crystallization rate of CF/PA6.

The crystallization process with a constant cooling rate (similar to the experimental temperature curve) for CF/PA6 is represented by the improved model, and the results are shown in Figure 4. Figure 4a,b illustrate the variations in relative crystallinity and crystallization rate, respectively. The black circles represent the experimental results, the red lines labeled “*X*-Model” in the legend represent the data obtained from the original values of *k* and *T*_s_ (parameters of a, b, c, and d are not used), and the dashed blue lines represent the results obtained with the fitted parameters of *k* and *T*_s_ when also employing the parameters of a, b, c, and d. Overall, the model results are in good agreement with the experimental results, successfully illustrating the non-isothermal crystallization of CF/PA6. The linear assumption of *K*(*T*) after crystallization onset ensures that the absolute value of the exponential term in Equation (8) remains sufficiently large to allow the relative crystallinity to reach 1.

As shown in Figure 4b, at the beginning of crystallization, there is a discrepancy between the predicted crystallization rate and the experimental data. This discrepancy is due to differences between the assumed function of *K*(*T*) and the experimental *K*(*T*) value at the onset of crystallization. In the latter half of the crystallization process, crystallization rate data of the model are initially higher and then slightly lower than the experimental data. This phenomenon can be explained by noting Equation (10), where *K*(*T*) increases as temperature decreases after crystallization begins (Figure 2), and the power function part decreases rapidly as *K*(*T*) increases. The physics of the real crystallization process is not involved here because the assumption of the model is that the function form of *K*(*T*) is based on the fitting of experimental data rather than the crystalline form during the microcrystallization process.

## 3. Verification of the Improved Model

### 3.1. Application of the Improved Model to Non-Isothermal Crystallization of PA6

**Figure 5:**
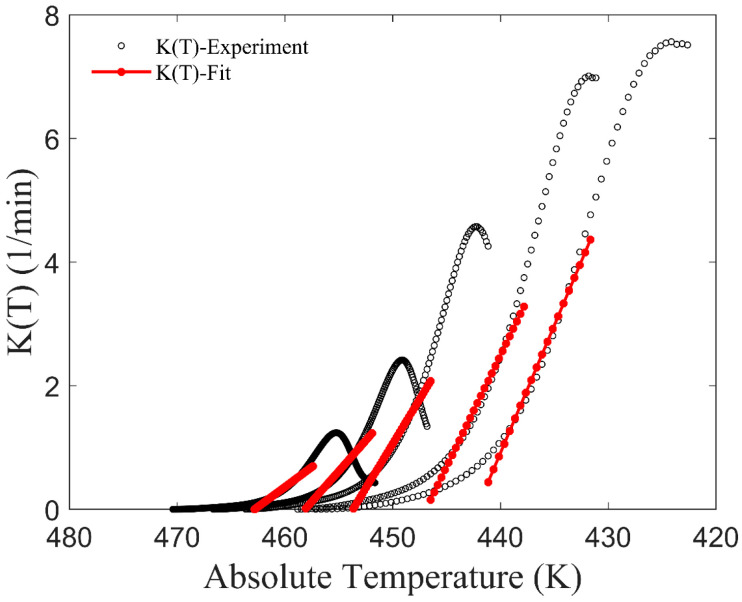
*K*(*T*) of PA6 in Mampel model and the fitted lines of a certain stage.

**Figure 6:**
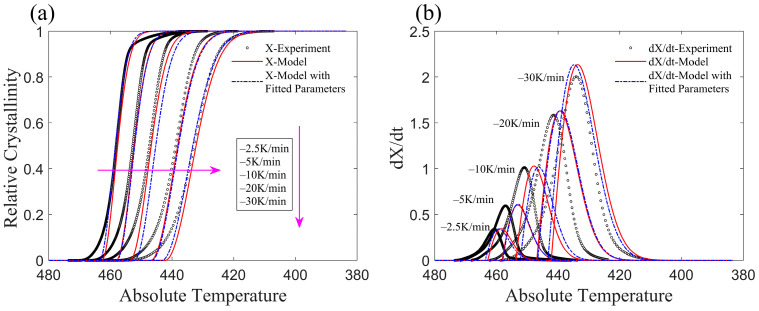
Comparison between model and experiment of PA6. (**a**) Relative crystallinity of PA6; (**b**) crystallization rate of PA6.

To expand the applicability of the improved model, the non-isothermal crystallization of PA6 was analyzed. As shown in Figure 5, there is no apparent linear relationship between *K*(*T*) and *T*. The relative crystallinity and crystallization rate derived from the revised model are shown in Figure 6. Since *K*(*T*) is assumed to be zero at the initial stage of crystallization, which significantly differs from the actual behavior of PA6, there is a marked discrepancy in the relative crystallinity at the onset of crystallization. However, when focusing on the primary crystallization stage (e.g., a relative crystallinity between 0.2 and 0.8), the model adequately describes the non-isothermal crystallization of PA6. The microscopic interfaces in CF/PA6 facilitate a rapid increase in *K*(*T*).

### 3.2. Application of the Improved Model to Published Experiments

Several published studies were selected to evaluate the applicability of the improved model across different materials. For accuracy, *K*(*T*) data within the relative crystallinity range of 0.1 to 0.6 for each material were selected for linear fitting.

(1) CF/PA6 (Fiber volume fraction 50%)

**Figure 7:**
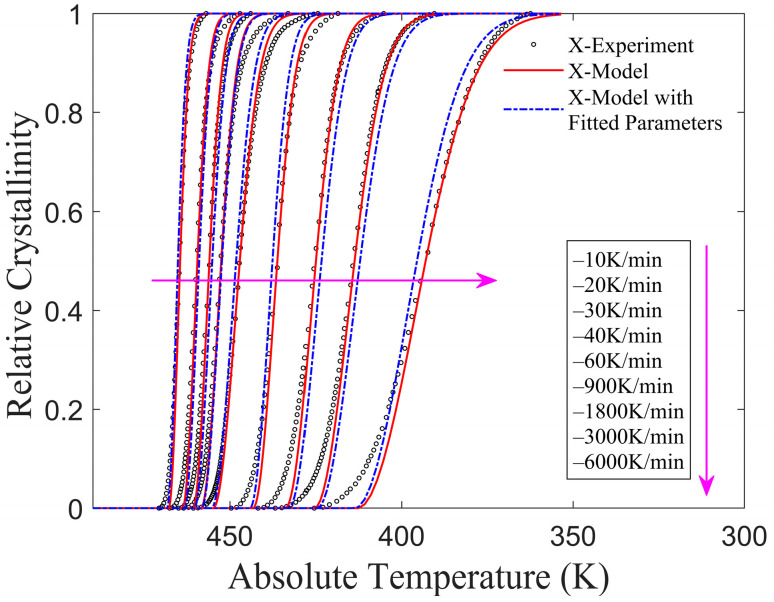
Relative crystallinity of CF/PA6 as determined by two methods.

**Figure 8:**
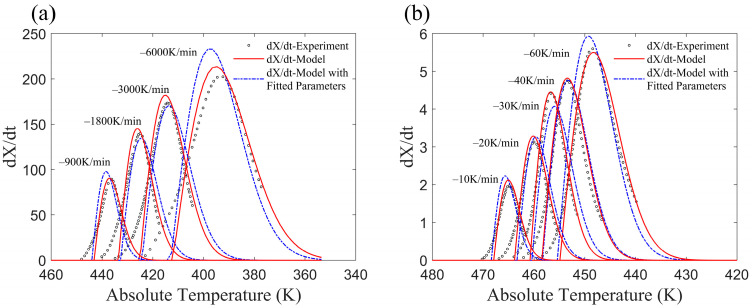
(**a**) Crystallization rate at high cooling rate; (**b**) crystallization rate at low cooling rate.

Kugele et al. [24] employed two methods to investigate the crystallization process over a wide range of cooling rates. Standard DSC was used to analyze the non-isothermal crystallization at a lower cooling rate, while flash DSC was employed for higher cooling rates. The relative crystallinities obtained from the revised model and experiment results are shown in Figure 7, and the crystallization rates obtained from the revised model as well as standard and flash DSC are shown in Figure 8. It can be found that the results from the revised model align closely with the experimental results, indicating that the non-isothermal crystallization of CF/PA6 composites, as reported in [24], can be accurately represented by this revised model.

(2) Other materials

**Figure 9:**
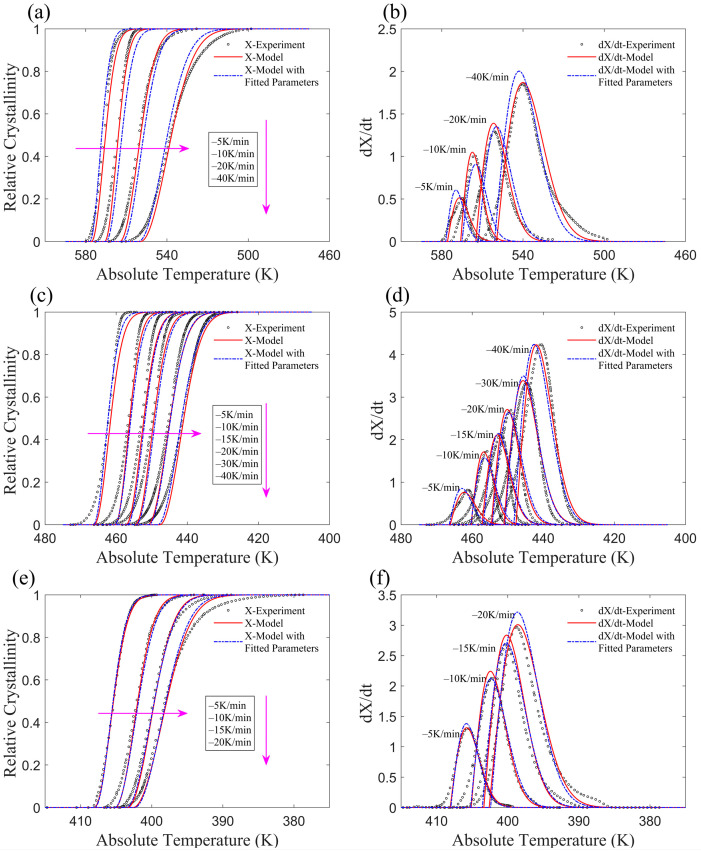
Comparison between models and experiments of several materials. (**a**) Relative crystallinity of SGF/PEEK composites; (**b**) crystallization rate of SGF/PEEK composites; (**c**) relative crystallinity of SCF/PTT composites; (**d**) crystallization rate of SCF/PTT composites; (**e**) relative crystallinity of PTFE/PP composites; (**f**) crystallization rate of PTFE/PP composites.

Yang et al. analyzed the non-isothermal crystallization of SGF/PEEK (short glass fiber-reinforced poly (ether ether ketone)) composites [38]. The relative crystallinity and crystallization rate illustrated by the revised model are shown in Figure 9a,b, which demonstrate strong agreement with the experimental results. Run et al. [15] examined the non-isothermal crystallization of SCF/PTT (short carbon fiber-reinforced Poly(trimethylene terephthalate)) composites. The relative crystallinity and crystallization rate represented by the revised model are shown in Figure 9c,d, which are consistent with the experimental results. Qiao et al. [22] investigated the non-isothermal crystallization of PTFE/PP (polytetrafluoroethylene reinforced polypropylene) composites. The relative crystallinity and crystallization rate depicted by the model are presented in Figure 9e,f, which agree well with the experimental results.

### 3.3. Assessment of Model Applicability

To evaluate the applicability of the model across different materials and to characterize the error of the revised model more intuitively, the absolute temperature differences between experimental and the revised model data were assessed at 9 points of relative crystallinity: 0.1, 0.2, 0.3, 0.4, 0.5, 0.6, 0.7, 0.8, and 0.9. The mean and maximum temperature differences were selected as the main parameters in order to quantify the error of the revised model. The specific form of the function is illustrated in Equation (13).

(13) $$\left\{\begin{array}{c} \Delta T_{mean}=mean\left(abs\left({\left.T^{experiment}\right|}_{X=0.1\times i}-{\left.T^{model}\right|}_{X=0.1\times i}\right)\right),\;i=1,2,\ldots ,9\\ \Delta T_{\max}=max\left(abs\left({\left.T^{experiment}\right|}_{X=0.1\times i}-{\left.T^{model}\right|}_{X=0.1\times i}\right)\right),i=1,2,\ldots ,9 \end{array}\right.$$

**Figure 10:**
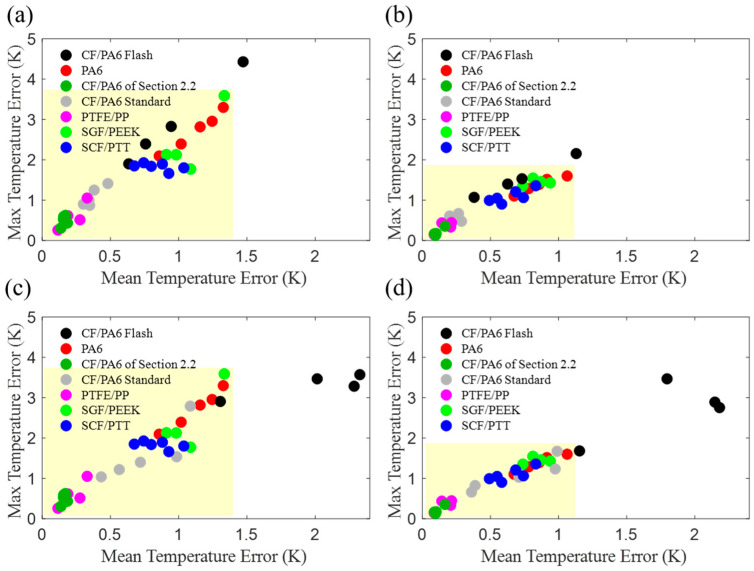
Error analysis. (**a**) Errors of model with origin parameters when *X* = 0.1, 0.2… 0.9; (**b**) errors of model with origin parameters when *X* = 0.2, 0.3… 0.8; (**c**) errors of model with fitted parameters when *X* = 0.1, 0.2… 0.9; (**d**) errors of model with fitted parameters when *X* = 0.2, 0.3… 0.8.

The model-calculated temperature errors at specific levels of crystallinity for different materials under various cooling rates are shown in Figure 10. The horizontal coordinate represents the average error, while the vertical coordinate signifies the maximum temperature difference.

As illustrated in Figure 10, the flash DSC (black dots) exhibits the greatest discrepancy, which is attributable to the rate of cooling being excessively rapid. This is attributed to the broader temperature range of the crystallization process, which can reach up to 60 K when cooling rapidly. However, in general, the error remains within an acceptable range.

If the flash DSC is not considered, as depicted in the yellow area of Figure 10a,c, the maximum errors of the revised model are all less than 4 K. For the range of relative crystallinity from 0.2 to 0.8 (yellow area of Figure 10b,d), the maximum errors of the revised model are all less than 2 K. Moreover, in the relative crystallinity range of 0.1–0.9, the average error is less than 1.4 K (yellow area of Figure 10a,c). In the 0.2–0.8 range, the average error is less than 1.1 K (yellow area of Figure 10b,d), which is comparable to the accuracy of standard thermocouples.

As illustrated in all four subplots in Figure 10, regardless of the flash DSC data, the model demonstrates a greater discrepancy in accurately depicting the crystallization process of the resin than that of the composites. Therefore, it can be concluded that the revised non-isothermal crystallization kinetics model with a piecewise function of *K*(*T*) is more suitable for application in thermoplastic composites than thermoplastic polymers.

## 4. Parameter Analysis of the Revised Mampel Model

**Figure 11:**
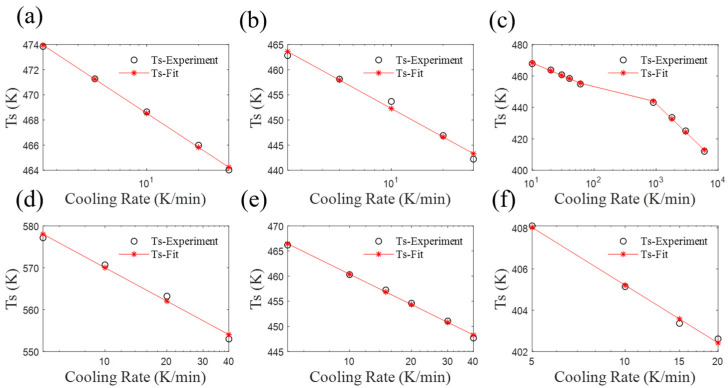
Variation of parameter *T*_s_ with cooling rate. (**a**) CF/PA6 in Section 2.2, (**b**) PA6, (**c**) CF/PA6 of reference, (**d**) SGF/PEEK, (**e**) SCF/PTT, and (**f**) PTFE/PP.

In the revised model, the slope *k* in the non-zero segment of *K*(*T*) and the initial crystallization temperature *T*_s_ are treated as functions of the cooling rate (Equation (12)); the values for other cooling rates, which are not tested with DSC, are then obtained through data fitting. As shown in Figure 11, the initial crystallization temperature *T*_s_ of various materials exhibits a well-defined relationship with the cooling rate. It is notable that the broken line in Figure 11c is a consequence of Kugele’s utilization of two test devices (standard DSC and flash DSC) for the purpose of testing [24]. Both data sets demonstrate that *T*_s_ has a strong linear correlation with log(*φ*). In fact, if the model is strictly applied within the experimental range ([*φ*_1_, *φ*_2_]), the relationship between *T*_s_ and log(*φ*) can be better fitted using a polynomial function.

**Figure 12:**
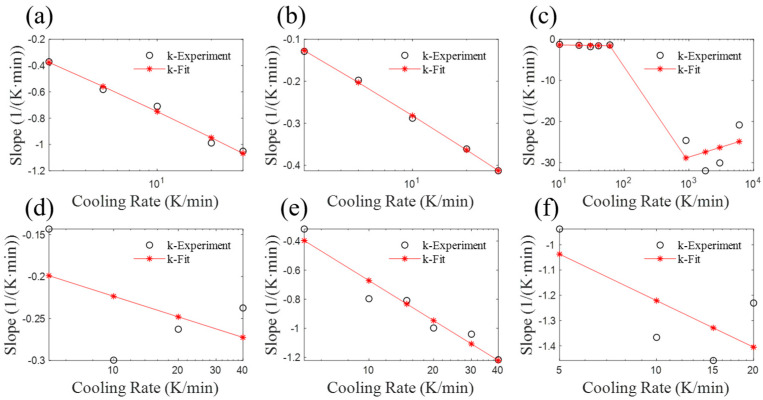
Variation of parameter *k* with cooling rate. (**a**) CF/PA6 in Section 2.2, (**b**) PA6, (**c**) CF/PA6 of reference, (**d**) SGF/PEEK, (**e**) SCF/PTT, (**f**) PTFE/PP.

The regularity of the parameter value *k* is relatively poor, but there is a general trend observable. It can be observed from Figure 12 that an increase in the cooling rate is accompanied by an increase in the absolute value of the slope. All the *T* (temperature)-*X* (relative crystallization) data obtained with fitted parameters are shown with blue dashed line in the corresponding figures above (Section 2 and Section 3). It is evident that the influence of the *k* value on the curve is relatively small. A comparative analysis of the relative crystallinity of SGF/PEEK in Figure 9a and parameter k in Figure 12d suggests that a 30% margin of error for *k* is acceptable.

## 5. Conclusions

In this work, the *K*(*T*) function in the Mampel model is described using a piecewise function with two parameters that can be easily obtained. The revised non-isothermal crystallization kinetics model with a piecewise function of *K*(*T*) can be applied to both composites and polymers and the initial proposed application of this method is on thermoplastic composites. The model initiates slightly later and completes crystallization earlier than the actual process, yet these deviations are within acceptable limits. At the start of crystallization, *K*(*T*) increases as the temperature decreases. Since this assumption is also applied in the later stage of crystallization, the model predicts the completion of crystallization earlier than it actually occurs. Although this results in some errors, compared to Ziabicki’s equation, this model ensures that the exponential terms in Equation (8) tend towards negative infinity and the final relative crystallinity of the material can be guaranteed to be 1. Additionally, this model does not utilize the Nakamura model, thus avoiding the need to obtain the Avrami index through isothermal testing, enhancing its convenience for engineering applications.

## Data Availability

The raw data supporting the conclusions of this article will be made available by the authors on request.
